# Physicochemical Properties of Nanoliposomes Encapsulating Grape Seed Tannins Formed with Ultrasound Cycles

**DOI:** 10.3390/foods13030414

**Published:** 2024-01-27

**Authors:** Angela Monasterio, Fernando A. Osorio

**Affiliations:** Department of Food Science and Technology, Technological Faculty, University of Santiago—Chile, USACH, Av. El Belloto 3735, Estación Central, Santiago 9170022, Chile; angela.monasterio@usach.cl

**Keywords:** tannins, antioxidants, nanoliposomes, ultrasound

## Abstract

Grape seeds are an excellent source of flavonoids and tannins with powerful antioxidant properties. However, the astringency of tannins limits their direct incorporation into food. To overcome this challenge, we investigated the encapsulation of grape seed tannins within nanoliposomes formed by ultrasound cycling. We characterized the nanoliposomes’ physicochemical properties, including encapsulation efficiency, antioxidant activity, stability, microstructure, and rheological properties. Our findings reveal that the nanoliposomes exhibited excellent stability under refrigerated conditions for up to 90 days with a mean particle size of 228 ± 26 nm, a polydispersity index of 0.598 ± 0.087, and a zeta potential of −41.6 ± 1.30 mV, maintaining a spherical multilamellar microstructure. Moreover, they displayed high antioxidant activity, with encapsulation efficiencies of 79% for epicatechin and 90% for catechin. This innovative approach demonstrates the potential of using ultrasound-assisted nanoliposome encapsulation to directly incorporate grape seed tannins into food matrices, providing a sustainable and efficient method for enhancing their bioavailability and functionality.

## 1. Introduction

Tannins constitute a distinctive category of phenolic compounds renowned for their remarkable antioxidant capacity and robust affinity for proteins and carbohydrates [1]. These molecules possess the ability to shield cells from oxidative stress induced by free radicals, which can have beneficial effects on health [2]. Recent research has unveiled the potential of tannins to safeguard the cardiovascular system by lowering blood pressure and enhancing blood circulation [3,4]. They can impede the production of pro-inflammatory substances in the body and induce apoptosis in cancer cells, rendering them intriguing natural bioactive molecules [5].

Grape seeds serve as an exceptional source of condensed tannins generated through the amalgamation of metabolic pathways involving shikimic acid and malonyl-CoA. This process yields flavan-3,4-diols (monomeric units) that subsequently polymerize through condensation reactions, forming grape seed proanthocyanidins. These proanthocyanidins primarily consist of units of (+)-catechin and (−)-epi-catechin and rank among the most prevalent compounds in grapes and wine [6]. Traditionally, the extraction of these compounds relied on nonenvironmentally friendly solvents such as dichloromethane, chloroform, and benzene derivatives [7]. In recent research, there has been a growing emphasis on exploring ultrasound as a prominent technique in the extraction process, driven by its remarkable efficiency [8]. This method has emerged as an effective tool for obtaining compounds of interest in various fields, ranging from the food industry to pharmaceuticals. An example of its effectiveness is observed in extracting flavors and essential oils from herbs and spices, where ultrasound has proven to be an efficient alternative to conventional methods [9]. Additionally, in the food industry, this approach has successfully extracted antioxidants from diverse sources, such as fruits and vegetables [10]. The efficacy of ultrasound lies in its ability to generate high-frequency sound waves, creating cavities and microcurrents in liquids, thus facilitating the efficient release and extraction of substances of interest [11]. This method has demonstrated speed and efficacy and offers sustainability benefits by decreasing dependence on harsh chemical solvents and elevated temperatures. In contrast to traditional extraction methods, it promotes an environmentally friendly approach. Ultrasound-assisted extraction strives for sustainable, eco-friendly, and efficient processes in extracting proanthocyanidins from grape seed powder [12]. This innovative technique distinguishes itself for its effectiveness and potential to enhance eco-conscious extraction practices, ultimately fostering more sustainable production processes within proanthocyanidins.

Despite their high antioxidant capacity, the incorporation of tannins into food is restricted due to their interaction with salivary proline, leading to an unpleasant astringent taste [13]. While the interaction mechanism of tannins with salivary proteins has been extensively investigated [14,15,16], their interaction with lipids has yet to be explored and is mainly limited to studies involving emulsions [17,18]. Nanoliposomes emerge as a promising solution for encapsulating tannins, preventing undesired reactions and enabling the introduction of the active ingredient into food without compromising flavor. Liposomes, spherical vesicles formed by a bilayer of phospholipids, can encapsulate hydrophobic and hydrophilic molecules [19]. They find wide applications in biological contexts due to their high biocompatibility with cell membranes, enabling the controlled release of the active ingredient within the organism [20]. Encapsulation within liposomes enhances the physicochemical properties of tannins, bolstering stability while preserving morphology and distribution [21]. Recent research indicates that the nano-incorporation of flavan-3-ols and proanthocyanidins from Merlot cultivar bagasse into liposomal vesicles maintains phenol stability for 30 days under refrigerated conditions without altering physicochemical properties, including antioxidant activity, morphology, and particle distribution [22]. Another study demonstrated the high biocompatibility between ellagitannins, proanthocyanidins, and flavonoids from pomegranate peel when encapsulated in lipid-based nanocarriers, which are also generally recognized as safe (GRAS) [23]. Once again, the versatility of ultrasound waves is revealed as they prove instrumental in extracting tannins and optimizing the physical characteristics of liposomes, such as size and polydispersity [24]. The application of ultrasound in tannin extraction enables the efficient release of these compounds, underscoring the utility of this technology in obtaining specific components with bioactive properties [25]. On the other hand, within the context of liposomes, ultrasound becomes a valuable tool for controlling uniformity in the size of these vesicles, as well as their distribution in the solution. This sonic approach demonstrates itself as an effective strategy for enhancing liposome quality. It highlights the versatility of ultrasonic waves in various biotechnological and pharmaceutical processes, establishing them as a multifaceted technique with promising applications in the engineering of liposomal systems [26].

Therefore, this study aims to characterize the physicochemical properties of nanoliposomes encapsulating grape seed tannins (TLS) formed through ultrasound cycles for their prospective incorporation into food. This endeavor seeks to unravel the mechanism of action of these compounds and propose a more sustainable and efficient approach to incorporating tannins into foods. This research explores the ecological and sustainable potential of ultrasound in extracting antioxidant molecules from grape residues, thus contributing to the valorization of agro-industrial waste. Furthermore, it underscores the nanoliposome encapsulation process as an effective and stable means of transporting and preserving bioactive compounds, preventing undesirable interactions. Ultimately, the characterization will provide crucial information about nanoliposome stability, morphology, and distribution, enhancing our understanding of the compounds’ mechanisms of action and facilitating precise and effective formulations for future biological applications.

## 2. Materials and Methods

### 2.1. Materials

Condensed tannin powder with a mean degree of polymerization of 2.5 ± 0.2, a gallotannin degree of 15.5 ± 1.1, and an average molecular weight of 784 ± 61, sourced from the Enology Laboratory of the Pontifical Catholic University of Chile, was used. Soy lecithin was acquired from Dimerco Comercial Ltda. (Santiago, Chile). Glycerol PA (purity ≥ 99%) was obtained from Sigma Aldrich (St. Louis, MO, USA). The 2,2′-azino-bis-3-ethylbenzothiazoline-6-sulfonic acid diammonium salt (ABTS) ≥98% and 2,4,6-tris(2-pyridyl)-s-triazine (TPTZ) were used for the FRAP assay, and Folin–Ciocalteu reagent were purchased from Sigma Aldrich (St. Louis, MO, USA). Solvents included PA-grade ethanol, HPLC-grade methanol (≥99.9%), trifluoroacetic acid (TFA) from Merck (Darmstadt, Germany), and Milli-Q water (PURELAB CLASSIC SYSTEM, ELGA LabWater, Lane End, UK).

### 2.2. Manufacture of TLS

The TLS was prepared using the heating/homogenization method with laminarity and size reduction through ultrasound cycles with some modifications [27]. To prepare 100 [mL] TLS [1 mg/mL], 0.1 g of grape seed powder was weighed on an analytical balance (Kern PFB, Ebingen, Germany) and mixed with 18 [mL] of sodium citrate/citric acid buffer (0.1 M at pH 3), and then 1 [g] of phosphatidylcholine (PC) was added to the mixture. It was stirred magnetically (DLab, MS-H280-PRO, Beijing, China) at 700 rpm for 5 [min]. The liposomal suspension was heated in a temperature-regulated bath (Julabo, ED (v.2), Seelbach, Germany) at 80 °C for 1 [h]. After this time, 0.76 g of glycerol as a lipoprotector [28], dissolved in 40 mL of buffer, was added and heated to 80 °C for one h. After this second heating period, the remaining 40 mL of buffer was added to achieve a 100 [mL] volume, and five vortex cycles (Equilab, Maxi mix II, Madrid, Spain) and ten ultrasound cycles were applied using an ultrasonic cell disruptor (HIELSCHER UP100H, Teltow, Germany, maximum 100 W) with an MS7 Micro-tip seven sonotrode (7 mm diameter, 120 [mm] length, 130 [W/cm^2^] acoustic power density) operating at 50% amplitude. Empty liposomes (LS) and tannins in suspension (TS) were used as controls. The samples were stored and refrigerated (4 °C) until characterization.

### 2.3. Encapsulation Efficiencies (EE)

The methodology described by Babazadeh [29] was used to determine the EE. A 2 mL sample was centrifuged at 4 °C and 14,000 rpm for 1 h (Hanil Scientific Inc. Supra R22, Incheon, Republic of Korea) to separate the nonencapsulated tannins. The supernatant was filtered using 0.22 μm syringe filters (Startech, New Taipei, Taiwan) and deposited into borosilicate vials (Finetech, New Taipei, Taiwan) for UHPLC analysis (Thermo Scientific Dionex UltiMate 3000, Waltham, MA, USA). For tannin analysis, a C18 column (5 μm, 250 × 4.6 mm, Perkin Elmer, Shelton, CU, USA) and a UV detector were used. The mobile phases and gradients used were described by Bianchi [30]. Catechin monomers were used as standards for tannin quantification, with calibration curves (5–500 μg/mL, R^2^ = 0.999). Monomer detection was performed at 280 nm. The EE was determined in triplicate and calculated using Equation (1).

(1) $$EE=\frac{{\left[T\right]}_{E}-{\left[T\right]}_{F}}{{\left[T\right]}_{E}}\times 100$$

where:

${\left[T\right]}_{E}$: initial concentration of encapsulated tannins, [mg catechin or epicatechin/g].

${\left[T\right]}_{F}:$ Free tannins in suspension [mg catechin or epicatechin/g].

### 2.4. Total Phenolic Content (TPC)

The quantification of TPC was performed according to the methodology described by Cano [31] with some modifications. Ten microliters of the sample were added to a semi-micro cuvette of a spectrophotometer, followed by 750 µL of distilled water. Fifty microliters of Folin–Ciocalteu reagent (Sigma Aldrich, No. CAS 10377-48-7, St. Louis, MO, USA) were added, mixed, and allowed to stand for 3 min. Then, 150 µL of a 7.5% (*w*/*v*) sodium carbonate solution was added, and the reaction proceeded for 1 h without light. Subsequently, absorbance at 760 nm was measured using a UV‒VIS spectrophotometer (Thermo Scientific, ORION AQUAMATE 8000, Waltham, MA, USA). TPC was calculated according to Equation (2).

(2) $$TPC=\frac{Abs\;}{m}=\left[GA\right]$$

where:

*Abs*: absorbance data recorded at 760 nm. 

*m*: Slope of the calibration curve performed with the gallic acid standard.

[*GA*] is the concentration of total phenols in gallic acid [mg/mL].

### 2.5. Total Anthocyanin Content (TAC)

The differential pH method was used to determine TAC, which is based on the structural change to the anthocyanin chromophore between pH 1 and 4.5 [32]. In 1.5 mL Eppendorf tubes, a 200 µL aliquot of the sample was added and diluted with 800 µL of a potassium chloride buffer solution (0.025 M) at pH 1. In parallel, in another Eppendorf tube, a 200 µL aliquot of the sample was added and diluted with 800 µL of a sodium acetate buffer solution (0.4 M) at pH 4.5. The reaction was carried out for 30 min at 20 °C without light. The absorbances of each tube were recorded in 1.5 mL semi-micro cuvettes at 520 nm and 700 nm using a UV‒VIS spectrophotometer (Thermo Scientific, ORION AQUAMATE 8000, Waltham, MA, USA). TAC was calculated using Equation (3).

(3) $$TAC=\frac{A\times PM\times FD\times 1000}{\varepsilon \times l}$$

where:

*TAC*: Total anthocyanin content expressed in [mg of cyanidin-3-glucoside/g of sample]. 

*A*: Change in absorbance of the sample at different pH values: $\left(Abs_{520}-Abs_{700}\right)pH1-\left(Abs_{520}-Abs_{700}\right)pH4.5$. 

MW: Molecular weight for cyanidin-3-glucoside = 449.2 [g/mol]. 

DF: Dilution factor used. 

*ε*: Molar extinction coefficient for cyanidin-3-glucoside = 26,900 [L/mol cm]; 

*l*: Cell path length = 1 [cm].

### 2.6. Total Tannin Content (TTC)

TTC was determined following the methodology of Corona [33] with some modifications. Two 15 mL Falcon tubes (A and B) were prepared, each containing 1 mL of diluted sample (FD = 10), 0.5 mL of distilled water, and 3 mL of concentrated hydrochloric acid (HCl) (37%). Tube A was heated to 90 °C for 30 min in a temperature-controlled bath (Julabo, ED (v.2), Germany), while tube B was kept at room temperature (20 °C). After the heating time, tube A was tempered until it reached 20 °C. Once tube A was tempered, 0.5 mL of absolute ethanol was added to each tube (A and B) to stop the reaction. The absorbance was measured at 280 nm using a UV‒VIS spectrophotometer (Thermo Scientific, ORION AQUAMATE 8000, Waltham, MA, USA). TTC was calculated using Equation (4).

(4) $$TTC=\left(Abs_{A}-Abs_{B}\right)\times X$$

where:

TTC: total tannin content [mg cyanidin-3-glucoside/g of sample].

${\mathrm{Abs}}_{A}$: Absorbance of tube A.

${\mathrm{Abs}}_{B}$: Absorbance of tube B.

X: Factor to express the results as mg cyanidin-3-glucoside/g = 1162.5.

### 2.7. Antioxidant Activity

#### 2.7.1. ABTS Assay

The ABTS radicals of the samples were evaluated following the methodology described by Sridhar and Charles [34], with some modifications. Before the assay, a stock solution of ABTS radical (7 mM ABTS in 2.45 mM K_2_S_2_O_8_) was prepared and kept in the dark for 16 h. After this period, an aliquot of the stock solution was diluted to an absorbance value of 0.7 ± 0.02 at 734 nm (adjusted ABTS reagent). A control was prepared by mixing 20 µL of ethanol with 980 µL of ABTS reagent. On the other hand, 20 µL of the sample was mixed with 980 µL of ABTS reagent, and the reaction was allowed to proceed in the dark at 20 °C for 10 min. After this time, the absorbance was measured at 734 nm using a UV‒VIS spectrophotometer (Thermo Scientific, ORION AQUAMATE 8000, Waltham, MA, USA). The results were expressed as milligrams of catechin per gram of sample.

#### 2.7.2. FRAP Assay

The FRAP was determined according to the methodology described by Alemán [35] with some modifications. In semi-micro cuvettes of 1.5 mL, 30 µL of sample was mixed with 90 µL of distilled water and 900 µL of FRAP reagent (composed of a mixture of 25 mL of sodium acetate buffer pH 3.6, 2.5 mL of 10 mM TPTZ solution, and 2.5 mL of a 20 mM iron chloride solution). The cuvettes were incubated at 37 °C for 30 min in a temperature-controlled bath (Julabo, ED (v.2), Seelbach, Germany). After the incubation period, the absorbance of the samples was measured using a UV‒VIS spectrophotometer (Thermo Scientific, ORION AQUAMATE 8000, Waltham, MA, USA) at 595 nm. The results were expressed as milligrams of catechin per gram of sample.

### 2.8. Stability Study of Nanoliposomes

The stability study was carried out only on the LS and TLS samples because the particle size of TS is outside the detection range of the equipment. However, for comparative purposes, a particle size value of 742.7 ± 5.30 nm was reported by our research group [36]. Mean particle size (MPS) and polydispersity index (PDI) analyses for each sample were performed over 90 days of storage at 4 °C using dynamic light scattering (DLS) with a backscattering angle of 173° and a refractive index of 1.330 for the medium and 1.334 for phospholipids (Zetasizer Nano ZS, Malvern Instruments, Malvern, UK). Zeta potential (ξ) was measured by DLS using the same Zetasizer Nano ZS equipment [37,38].

### 2.9. Microstructure

#### 2.9.1. Transmission Electron Microscopy (TEM)

The microstructure of TLS was studied using TEM with a Talos F200C G2 microscope (Thermo Scientific, Waltham, MA, USA). Negative staining was applied to enhance contrast. Samples were deposited onto a copper grid coated with Formvar/Carbon (300 mesh, 3 mm diameter HF 36). Then, a 2% *w*/*v* uranyl acetate solution was added to the samples and they were allowed to sit for 1 min. Afterward, the grids were air-dried in an oven (LabTech model LDO-150F, Incheon, Republic of Korea) at 25 °C for 10 min [39].

#### 2.9.2. Cryogenic Transmission Electron Microscopy (Cryo-TEM)

For the structural analysis of TLS using cryo-TEM, perforated carbon grids Quantifoil R2/2 were employed with cryo-plunging at −180 °C. Vitrification was achieved using 3 µL of sample per grid within an 8–10 s immersion interval in liquid ethane [40]. Micrographs were captured using a JEOL JEM-1230 transmission electron microscope operating (Akishima, Tokyo, Japan) at 100 kV with a nominal magnification of 40 K, which was implemented with the aid of a Gatan 626 cryo-sample holder and coupled to a 4 K × 4 K TVIPS CMOS camera (TemCam-F416).

### 2.10. Rheological Properties

To determine the rheological properties of the samples, a rheometer (Discovery Hybrid Rheometer HR2, TA Instruments, New Castle, DE, USA) was employed with a cone-and-plate configuration (1008°; 60 mm in diameter and 27 µm gap between the geometry and the plate). The bottom plate was equipped with a Peltier temperature control system. One milliliter of sample was deposited on the bottom plate and stabilized for 5 min at the initial working temperature. An oscillatory amplitude test was conducted to determine the linear viscoelasticity range of each sample. Once this zone was determined, an upward temperature sweep from 0 °C to 80 °C and a downward sweep from 80 °C to 0 °C were performed at a heating/cooling rate of 2 °C/min, 1% strain, and 1 Hz angular frequency [41].

### 2.11. Statistical Analysis

All assays were performed in triplicate, and the experimental data obtained were expressed as the mean ± standard deviation. Differences between three or more groups were evaluated using one- or two-way ANOVA tests, followed by Tukey’s post hoc comparisons with a confidence level of 95% (*p* < 0.05) to determine statistical significance. All statistical analyses were conducted using STATGRAPHICS Centurion XVI software, v.16.1.03 (StatPoint Technologies, Inc., Warrenton, VA, USA) [42].

## 3. Results

### 3.1. Encapsulation Efficiency (EE)

Table 1 shows the results of the EE for catechin and epicatechin monomers within nanoliposomes with ultrahigh-performance liquid chromatography (UHPLC). The data are expressed as the mean values with corresponding standard deviations.

The results indicate that the nanoliposomes encapsulating grape seed tannins formed with ultrasound cycles exhibit an encapsulation efficiency of 87 ± 0.01% for catechin and 75 ± 0.02% for epicatechin. Different letters (a and b) within the same row signify statistically significant differences (*p* < 0.05) between the encapsulation efficiencies of catechin and epicatechin within the nanoliposomes.

These data underscore the effectiveness of the ultrasound-assisted formation process in achieving varying encapsulation efficiencies for distinct monomers, providing valuable insights into the encapsulation characteristics of grape seed tannins within nanoliposomal structures.

### 3.2. Antioxidant Activity

The antioxidant activity of the various samples, including TS, LS, and TLS, is detailed in Table 2. The outcomes of the analysis highlight significant differences (*p* < 0.05) among these samples.

TS stands out, with the highest TPC, TAC, and TTC levels. Additionally, it exhibits robust antioxidant activity regarding ABTS and FRAP assays. In contrast, LS demonstrates markedly lower antioxidant properties across all evaluated parameters. TLS falls between TS and LS, showcasing intermediate antioxidant activity.

The variations in antioxidant potential among the samples underscore these materials’ diverse health benefits and potential applications. Table 2 presents a comprehensive overview of the antioxidant activity, providing mean values and standard deviations for each parameter. The different letters in the same column indicate statistically significant differences (*p* < 0.05) between the samples.

This detailed analysis of antioxidant activity provides a comprehensive understanding of the distinctive properties of each sample, contributing valuable insights into their potential food applications.

### 3.3. Stability Study of TLS

**Figure 1:**
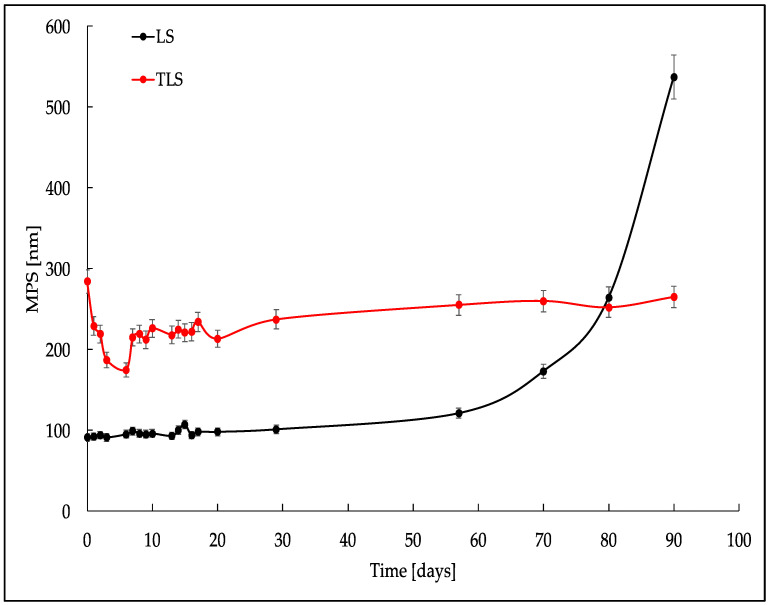
Mean particle size of nanoliposomes formed with ultrasound cycles during refrigerated storage.

**Figure 2:**
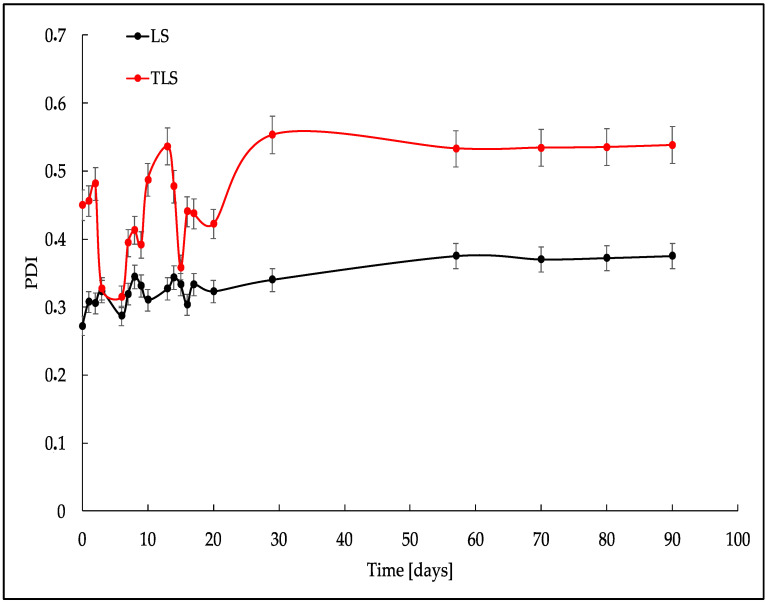
Polydispersity index of nanoliposomes formed with ultrasound cycles during refrigerated storage.

**Figure 3:**
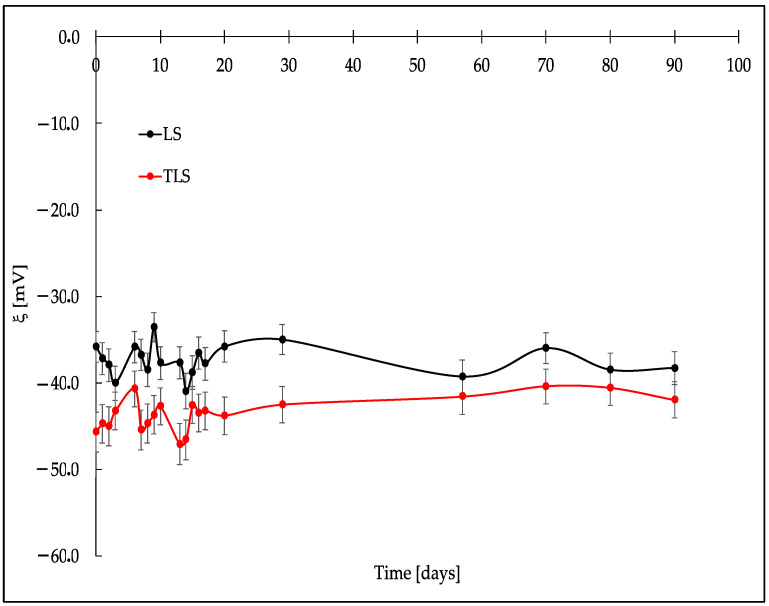
Zeta potential of nanoliposomes formed with ultrasound cycles during refrigerated storage.

Figure 1, Figure 2 and Figure 3 show a crucial stability study on the behavior of three key parameters: MPS, PDI, and ξ during 90 days of storage at 4 °C.

Figure 1 shows how the particle size changes, reflecting particle dispersion or aggregation alterations in the samples. The MPS, representing nanoparticle size, demonstrated dynamic changes in sample LS over the 90-day observation period, ranging from 91 to 537 nm. These fluctuations suggest potential variations in nanoparticle aggregation or dispersion, emphasizing the nanomaterial’s temporal sensitivity. Similarly, in the case of TLS, the MPS exhibited variability, spanning from 284 to 265 nm, indicating dynamic changes in nanoparticle size and providing crucial insights into the dynamic behavior of the nanomaterial.

The polydispersity index, indicative of particle size distribution uniformity, showed variability in both samples (Figure 2). For LS, the PDI ranged from 0.272 to 0.375, indicating changes in the homogeneity of particle sizes over time. Likewise, TLS displayed fluctuations in PDI from 0.45 to 0.375, providing critical information about the stability and uniformity of the nanoparticle system. Lower PDI values suggest a more homogeneous distribution. In comparison, higher values imply a broader range of particle sizes, as is the case for TLS, which presented significantly larger distributions than LS, mainly attributed to tannins.

Figure 3 indicates variations in the electric potential at the particle surfaces, which is essential for evaluating colloidal stability.

Regarding zeta potential, a measure of surface charge and colloidal stability, sample LS exhibited fluctuations between −35.8 and −38.3 mV. These variations in ξ provide insights into the electrostatic interactions within the nanoparticle system, contributing to the understanding of colloidal stability. TLS also displayed changes in ξ, ranging from −45.7 to −38.3 mV, highlighting alterations in surface charge and colloidal stability over the 90 days.

In summary, the comprehensive analysis of MPS, PDI, and ξ for LS and TLS samples offers valuable insights into the dynamic behavior of these nanoparticles. These results contribute essential information for understanding their stability, size distribution, and surface charge characteristics, thus advancing the knowledge base for potential applications in various scientific fields.

### 3.4. Microstructure

**Figure 4:**
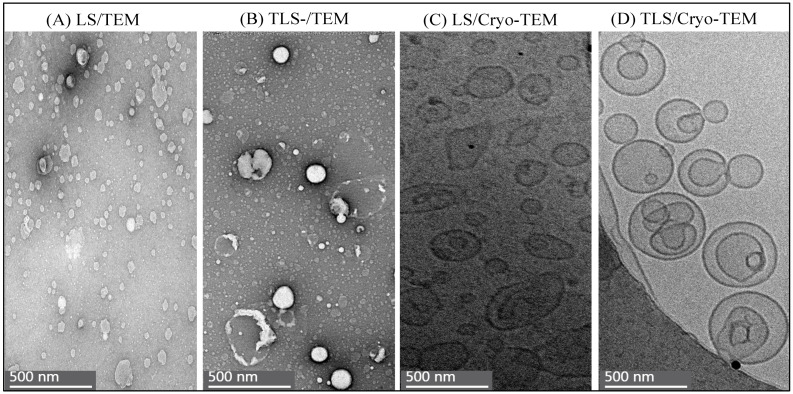
Distribution of LS and TLS obtained with TEM and Cryo-TEM. (**A**) LS/TEM; (**B**) TLS-/TEM; (**C**)LS/Cryo-TEM; (**D**) TLS/Cryo-TEM

**Figure 5:**
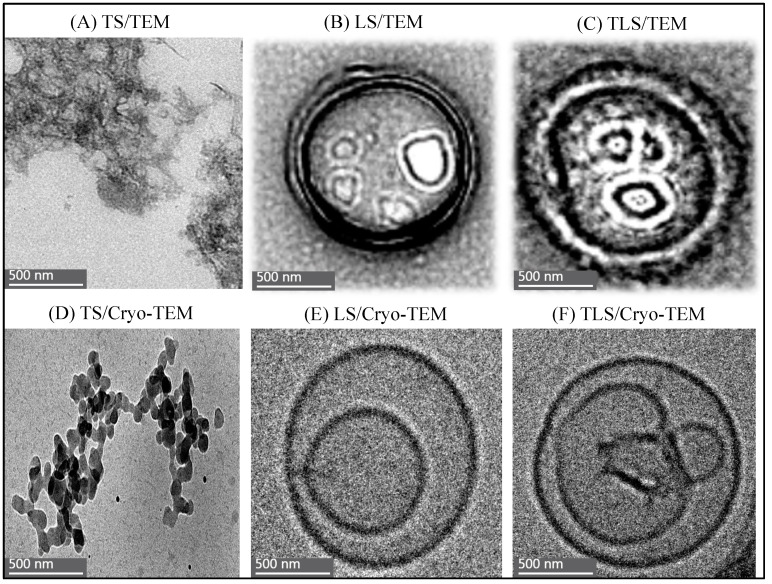
Micrographs obtained using TEM and Cryo-TEM. (**A**) TS/TEM; (**B**) LS/TEM; (**C**) TLS/TEM; (**D**) TS/Cryo-TEM; (**E**) LS/Cryo-TEM; (**F**) TLS/Cryo-TEM.

Figure 4 and Figure 5 offer valuable insights into the nanoscale morphology of the studied samples, as observed through TEM and Cryo-TEM.

Figure 4 displays the distribution of LS and TLS as captured by TEM and Cryo-TEM. These images provide a visual representation of these nanoparticles’ spatial arrangements and dispersion, offering a glimpse into their structural characteristics and the colloidal dispersion formed through the application of ultrasound cycles.

Figure 5 presents micrographs captured using TEM and Cryo-TEM. These high-resolution images showcase the detailed microstructure of the LS and TLS samples, allowing for a closer examination of their nanoscale features. These visual observations are crucial for understanding the physical properties and structural integrity of the materials under investigation and providing information on the location of bioactive compounds in the bilayer.

### 3.5. Rheological Properties

**Figure 6:**
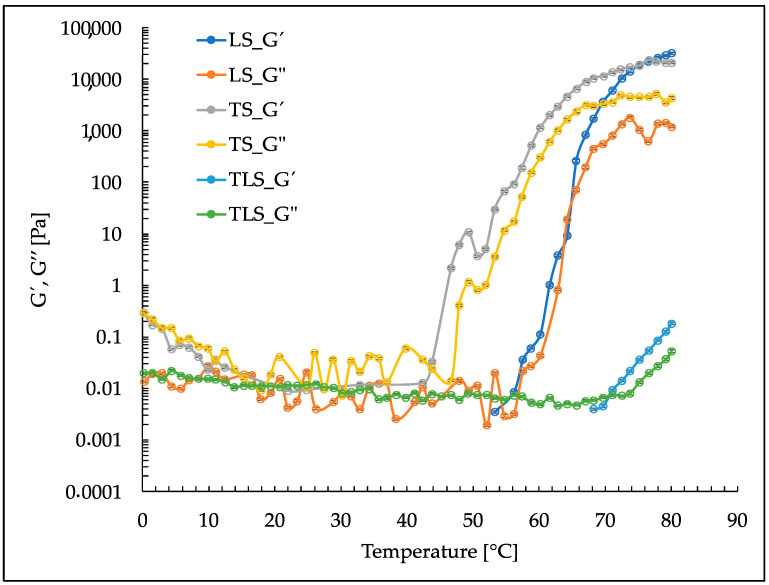
Upward temperature scans from 0 °C to 80 °C for TS and LS and TLS.

Figure 6 graphically demonstrates the upward temperature scan illustrating the variations in storage modulus (G’) and loss modulus (G”) as the temperature increases from 0 °C to 80 °C. These moduli provide critical insights into the materials’ ability to store and dissipate energy under varying thermal conditions.

The LS sample presented a dynamic viscoelastic behavior in the range of temperatures studied. At lower temperatures, specifically 53.33 °C, G’ is relatively low, 0.0035 [Pa], indicating weak resistance to deformation, while G” is 0.0135 [Pa]. As the temperature increases, both modules show an increasing trend. The transition from a predominantly elastic response to a more viscous behavior becomes evident as G’ exceeds G”. This trend continues, with a substantial increase in moduli at 75.19 °C, indicating a more pronounced solid-like response. Interestingly, at 80.06 °C, both moduli show a noticeable decrease compared to the previous temperatures, possibly indicating a transition toward more fluid behavior.

The rheological analysis of sample TS provides insights into its viscoelastic behavior. Contrasting responses at different temperatures further highlight the temperature-dependent behavior of TS. For instance, at 46.69 °C, G’ becomes significantly higher, suggesting a more solid-like response. Conversely, at 60.23 °C, the dominance of G” indicates a more liquid-like behavior. These variations in elastic and viscous properties showcase the intricate nature of TS rheological characteristics. Moreover, the rheological analysis of the TLS sample reveals that at lower temperatures, such as 68.31 °C, the material exhibits a G’ of 0.0039 [Pa], indicating a predominantly elastic response. As the temperature increases, there is a gradual transition toward more viscous behavior, as seen in the corresponding G’ increase. When examining specific temperatures, the response of the material varies. At 71.12 °C, there is a peak in G’, suggesting more solid-like behavior. In contrast, at 76.61 °C, G” dominates, indicating a shift toward liquid-like properties. These variations highlight the nuanced rheological characteristics of the TLS sample.

**Figure 7:**
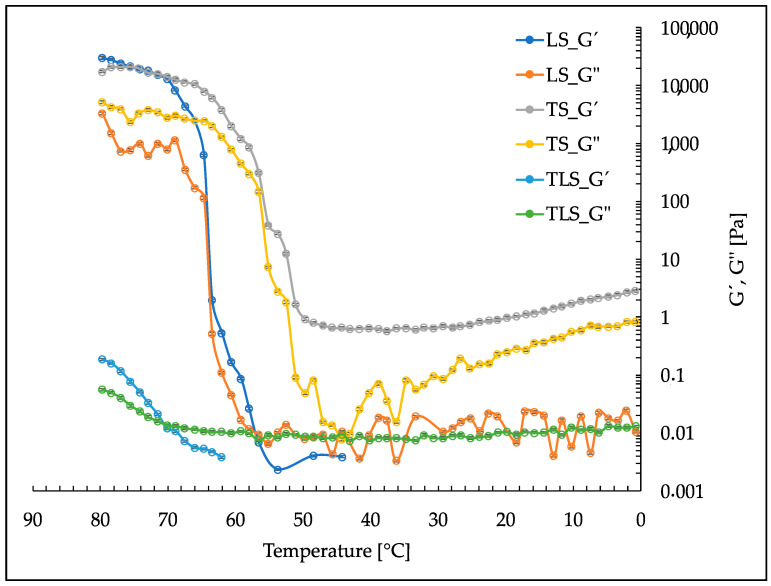
Downward temperature scans from 80 °C to 0 °C for TS and LS and TLS.

In contrast, Figure 7 depicts the results of a downward temperature scan, showcasing the dynamic changes in G’ and G” as the temperature is lowered from 80 °C back to 0 °C. These data shed light on the reversible behavior of the samples, particularly their viscoelastic properties with temperature fluctuations.

These rheological analyses are pivotal in understanding how the materials respond to temperature variations, providing fundamental information for various applications, such as formulation and processing considerations in the food industry.

## 4. Discussion

### 4.1. Encapsulation Efficiencies (EE)

The observed differences in EE can be attributed to the distinctive chemical and physical properties of the organic acid used in conjunction with sodium citrate in preparing the buffer, which serves as a tannin dispersion medium. Citric acid, a tricarboxylic acid, presents itself as a crystalline solid under normal conditions and exhibits high solubility in water. This specific molecular structure is likely to play a pivotal role in forming encapsulation complexes with catechin and epicatechin compounds [43]. The crystallinity of citric acid and its high solubility in water are key attributes that facilitate the formation of encapsulation complexes with catechin and epicatechin. This process is essential for encapsulation efficiency, as it establishes the basis for molecular interactions between the bioactive compounds and the liposomal matrix [44]. Moreover, the mechanical forces generated by ultrasound likely facilitated a more uniform dispersion of tannins within the liposomal matrix. This enhanced dispersion could be linked to the observed improvements in encapsulation efficiency [45].

The discerned disparity in encapsulation efficiency between catechin and epicatechin could be linked to subtle structural variations between these monomers. Both molecules feature two aromatic rings (A and B) with hydroxyl groups (-OH) attached. In the case of epicatechin, the hydroxyl group is situated at position 3, whereas in catechin, this hydroxyl group is found at position 4. This nuanced structural difference may have multifaceted effects on their chemical properties, including antioxidant activity, as well as their physical properties, such as solubility, molecular interactions with the encapsulation matrix, size, and three-dimensional shape. Moreover, the ultrasonic treatment could have further influenced the specific structural variations between monomers, particularly the position of the hydroxyl group in the aromatic rings. The cyclical application of ultrasound might have induced subtle changes in the molecular interactions between the tannins and the liposomal matrix, contributing to the observed differences in encapsulation efficiency. These factors collectively contribute to the encapsulation efficiency [46].

The use of ultrasound cycles introduces an additional dimension to the encapsulation process. The mechanical vibrations induced by ultrasound waves during liposome preparation can influence liposome size, stability, and overall structure, thereby affecting their ability to encapsulate bioactive compounds. In our study, the application of ultrasound cycles played a crucial role in enhancing the encapsulation efficiency of both catechin and epicatechin within the nanoliposomes [47].

The findings indicating a more effective encapsulation of catechin in nanoliposomes than epicatechin align with previous studies. These studies reported similarly high encapsulation efficiencies for nanoliposomes loaded with other bioactive compounds, such as α-linolenic acid (ALA), an ω-3 fatty acid, and polyphenols such as naringin and naringenin [48,49]. This consistency in results suggests that the nanoliposomal encapsulation system with ultrasound cycles, when coupled with the specific properties of these bioactive compounds, consistently leads to high encapsulation efficiencies. The combination of properties of the encapsulation material, structural variations of the bioactive compounds, and the application of ultrasound emerge as an interrelated set of critical factors determining encapsulation efficiency. These findings contribute to the fundamental understanding of encapsulation processes and provide a solid foundation for future research aimed at refining the formulation of nanoliposomes and their application in the efficient delivery of bioactive compounds.

### 4.2. Antioxidant Activity

The comparison of antioxidant activity between samples revealed differences in TPC, TAC, and TTC and underscored the influence of ultrasound cycles applied during nanoliposome formation.

The TS sample exhibited significantly higher TPC (43 ± 0.03 [mg GA/g]), TAC (13.86 ± 2.60 [mg cyd-3-glu/100 mL]), and TTC (24.84 ± 2.32 [mg cyd/100 mL]) values than the TLS sample. This observation aligns with expectations, considering that tannins in the tannin suspension are present in a free state, contributing to higher measured contents. These findings resonate with previous studies on nanoliposomes loaded with rutin [50].

Notably, the TS sample also demonstrated significantly higher antioxidant activity than the TLS sample, as determined by both ABTS and FRAP assays. The observed differences can be attributed to the encapsulation of tannins within the liposomal structure during ultrasound-assisted preparation. In liposomes, the active compounds are shielded within the structure, limiting their direct interaction with the ABTS radical and ferric ions, and consequently reducing the measured antioxidant activity. This phenomenon is consistent with the literature and highlights the importance of considering the encapsulation matrix when evaluating the antioxidant potential of compounds.

The application of ultrasound cycles introduces a nuanced aspect to these findings. While ultrasound can enhance the dispersion and encapsulation of bioactive compounds, it may also impact their structural integrity and, consequently, their antioxidant activity. In the context of this study, the cyclic application of ultrasound likely influenced the formation of nanoliposomes, affecting the interaction between tannins and the encapsulation matrix.

Furthermore, the lower values of antioxidant activity observed in the TS sample could indicate the trade-off between immediate antioxidant effects and the potential advantages of liposomal encapsulation, such as controlled release and stability. This trade-off underscores the need for a balanced approach when utilizing nanoliposomes to deliver antioxidants.

Finally, the antioxidant activity values obtained for the TLS sample are similar to those previously reported for nanoliposomes made with quercetin and liposomes made with hemp seed oil [51,52]. The complex interplay between liposomal encapsulation, multi-lamellarity associated with ultrasound cycles, and antioxidant activity of tannins highlights the need for comprehensive evaluation when designing nanoliposome delivery systems for antioxidant compounds, providing a solid foundation for future research and practical applications in the development of efficient delivery strategies for bioactive compounds with antioxidant properties.

### 4.3. Stability Study of TLS

The observed differences in the MPS between LSs and TLSs on day zero, with TLSs exhibiting larger sizes (284 ± 4 nm), can be attributed to the encapsulation of tannins. Tannins, characterized by large and nonuniform structures, are prone to aggregate, leading to colloidal dispersions with varying hydrodynamic diameters ranging from nanometers to microns [53]. The significant reduction in MPS observed in the TLS sample, highlighting a surface area/volume ratio improvement, aligns with findings suggesting that this solvent configuration enhances the extraction efficiency of compounds [54], which is consistent with the higher encapsulation efficiency (EE) observed for catechin and epicatechin monomers in the TLS sample.

The initial variability in MPS values observed during the first 20 days of refrigerated storage across all samples can be attributed to the formation, growth, and collapse of microbubbles generated by cavitation during ultrasound-assisted nanoliposome formation. The release of high-pressure and high-speed energy during this process contributes to structural changes, impacting particle size [55,56].

From days 21 to 90, the size of the TLS stabilized at an average of 228 ± 26 nm, demonstrating the long-term colloidal stability of the tannin-loaded liposomes. In contrast, the progressive increase in MPS observed in LS starting from day 60 suggests potential lipid membrane degradation and the formation of degradation products [57,58]. This prolonged stability suggests that the surface activity of tannins, particularly tannic acid, plays a pivotal role in maintaining the liposome size for an extended period under refrigeration conditions. In the context of liposomal stability, the amphipathic nature of tannic acid likely facilitates its adsorption at the liposome–water interface. This adsorption, akin to the behavior of surfactant molecules, contributes to the reduction in surface tension, stabilizing the liposomal structure over an extended storage period [59].

Regarding the polydispersity index, LS exhibited the narrowest particle size distribution on day zero (PDI = 0.272 ± 0.038), indicative of a homogeneous and monodisperse distribution. Incorporating a tannin suspension into liposomes increased PDI, and the TLS sample showed variability up to day 20, corresponding to changes in particle size in suspension [60]. However, similar to MPS, the PDI remained uniform from day 21 to 90, emphasizing the sustained stability of the tannin-loaded liposomes.

The surface charge, represented by the zeta potential, was highly negative for both LS (−35.8 ± 1.5 [mV]) and TLS (−45.7 ± 1.6 [mV]) at the beginning of the study. Throughout the refrigerated storage period, ξ values remained stable, with both samples exhibiting values less than −30 [mV], indicative of favorable conditions for maintaining liposomal stability due to repulsive forces that hinder aggregation or sedimentation [61]. Notably, the TLS sample had the most negative ξ value, suggesting higher colloidal stability, as particles are more dispersed and less prone to aggregation. In summary, the sonication technique, as applied in the formation of nanoliposomes, contributed to the colloidal stability of the particles. The encapsulation of tannins not only influenced the antioxidant activity and structural stability but also played a crucial role in maintaining the colloidal stability of liposomes over an extended period, reinforcing the potential of tannin-loaded liposomes for various food applications.

### 4.4. Microstructure

The microscopic examination of LS and TLS using electron microscopy techniques provides valuable insights into the sonication technique’s contribution to the particles’ colloidal morphology.

Figure 4A,C illustrate that LS shows a more uniform distribution than TLS, as seen in Figure 4B,D. The latter exhibits a skewed distribution toward the grid’s edges, attributed to its larger particle size, equal to or greater than 500 nm. This less homogeneous distribution of TLS is consistent across both TEM and Cryo-TEM, emphasizing that the observed differences are not dependent on the imaging technique. These findings align with the stability study results, where LS exhibited lower MPS and PDI values than TLS (Figure 1 and Figure 2).

The irregular morphology of the TS is evident in Figure 5A,D, revealing clusters of tannins. This characteristic structure demonstrates the inherent ability of tannins to form aggregates and complex structures [62].

Upon magnifying the micrographs in Figure 5, both LS (B and E) and TLS (C and F) exhibit the characteristic spherical morphology of vesicles. The presence of a double membrane formed by the polar heads and hydrophobic tails of phospholipids is evident in both samples [63]. Additionally, the observation of multi-lamellarity in the liposomes, represented by concentric lipid layers surrounding the aqueous cavity, suggests the impact of ultrasound cycles applied during their formation [64,65]. As observed in this study, multi-lamellar liposomes are less common than uni-lamellar liposomes and tend to have larger sizes and a higher capacity for loading compounds [66]. Furthermore, the multiple layers of TLS compared to LS slow the release of the active ingredient, which translates into lower antioxidant activity, so the results are related. Similar structural features were reported for nanoliposomes formed using canola lecithin to encapsulate Berberis vulgaris anthocyanins with a combined hydration and ultrasound method [67].

These micrographs offer a visual estimation of the nanostructures and potential localization of tannins. Denser structures interact more strongly with the electron beam, resulting in images with higher contrast [68]. The presence of tannins within the liposomal structure is suggested by the observed clustering in TS and the overall morphology of vesicles in both LS and TLS.

In summary, the electron microscopy findings complement the stability study results, providing visual evidence of the impact of the sonication technique on the colloidal stability of liposomes. The observed structural features and distribution patterns emphasize the role of ultrasound cycles in shaping the morphology and stability of the nanoliposomes, contributing to their potential for effective encapsulation and delivery of bioactive compounds such as tannins.

### 4.5. Rheological Properties

The rheological behavior observed in TS, LS, and TLS provides valuable insights into the contributions of ultrasound cycles to these formulations’ thermal and mechanical properties.

For TS, an increase in both the storage modulus (G’) and loss modulus (G”) was noted from 45 °C onwards. This phenomenon is attributed to the formation of aggregates or complexes, inducing an increase in viscosity and modifying the rheological behavior of the sample [69,70]. Similarly, LS exhibited a comparable increase in moduli at a temperature of 52 °C, possibly explained by a phase transition from the liquid to the gel state in the lipid membranes, affecting their permeability [71].

Conversely, TLS displayed an increase in G’ and G” from 65 °C onwards, attributed to interactions between the gallic acid fractions present in tannins and the phosphate groups of PC [72]. This rheological behavior is reminiscent of patterns reported for cross-linked coacervates with tannic acid and tannin foams [73].

In the food industry, nanoliposomes play a crucial role, but undesired thixotropy, i.e., changes in their viscoelastic properties over time, should be avoided. Figure 6 exhibits a similar behavior of G’ and G” with decreasing temperature for TS, LS, and TLS, indicating the absence of thixotropy. Tannins, a group of common polyphenols in plants, have emerged as critical agents in preventing thixotropy and stabilizing nanoliposomes [74], which is attributed to their specific properties, including the ability to form bonds with molecules on the surface of nanoliposomes and the formation of complexes (as observed between gallic acid fractions of tannins and phosphate groups in lipid membranes). These properties ensure that nanoliposomes maintain consistent mechanical and rheological properties over time, a critical factor in the formulation of processed foods to ensure the consistent quality of the final product [75,76].

These results provide insights into the complex interactions between tannins and nanoliposomes and their thermal behavior, which could subsequently influence their application as ingredients in processed foods. The rheological stability imparted by tannins, especially in TLS, highlights the potential for their use in the formulation of food products where consistent texture and mechanical properties are essential for product quality and consumer acceptance.

## 5. Conclusions

This study has validated the effectiveness of ultrasound cycles in the process of extracting proanthocyanidins from grape seed powder and their subsequent encapsulation in nanoliposomes, referred to as TLS. Comprehensive evaluations of physicochemical properties were conducted, addressing aspects such as the stability, microstructure, rheology, encapsulation efficiency, and antioxidant activity of TLS. The type of solvent used in tannin extraction and the application of ultrasound cycles showed a significant impact on both the total tannin content (CTT) and antioxidant activity, with values of 95 ± 0.82 [mg cyd-3-glu/g] and 0.5 ± 0.02 [mg catechin/g], respectively. The encapsulation efficiency was 87% for catechin and 75% for epicatechin. These subtle disparities were attributed to modifications in the structural conformation of the monomers induced by sonication.

It is noteworthy that TLS maintained its multilayered spherical structure with remarkable polydispersity and constant electric charge over 90 days of refrigerated storage. Additionally, no alterations were observed in its viscoelastic properties across the wide temperature range of 0 to 80 °C, affirming its suitability for application in various processed food categories. In summary, this research provides a comprehensive understanding of the interaction between tannins and nanoliposomes, opening a promising avenue for the controlled delivery of bioactive compounds in pharmaceutical and food contexts. 

## Figures and Tables

**Table 1:** Results EE of TLS using UHPLC *.

Sample	Catechin [%]	Epicatechin [%]
TLS	87 ± 0.01 ^a^	75 ± 0.02 ^b^

* Results are presented as the means ± standard deviations. Different letters in the same row indicate significant differences (*p* < 0.05). TLS: Nanoliposomes encapsulating grape seed tannins formed with ultrasound cycles.

**Table 2:** Antioxidant activity of TS, LS, and TLS *.

Sample	TPC [mgGA/g]	TAC [mg cyd-3-glu/g]	TTC [mg cyd-3-glu/g]	ABTS [mg Catechin/g]	FRAP [mg Catechin/g]
TS	43 ± 0.03 ^c^	137 ± 2.60 ^b^	248 ± 2.32 ^c^	0.2 ± 0.01 ^b^	2.2 ± 0.01 ^c^
LS	5 ± 0.01 ^a^	9 ± 0.35 ^a^	19 ± 0.00 ^a^	0.1 ± 0.01 ^a^	0.3 ± 0.01 ^a^
TLS	18 ± 0.02 ^b^	17 ± 2.60 ^a^	95 ± 0.82 ^b^	0.1 ± 0.01 ^a^	0.5 ± 0.02 ^b^

* The results correspond to the means ± standard deviations. Different letters in the same column indicate significant differences (*p* < 0.05). TS: tannin suspensions; LS: empty nanoliposomes; TLS: nanoliposomes encapsulating grape seed tannins formed with ultrasound cycles; TPC: total phenolic content; TAC: total anthocyanin content; TTC: total tannin content; ABTS: 2,2’-azino-bis-3-ethylbenzothiazoline-6-sulfonic acid diammonium salt; FRAP: ferric reducing antioxidant power.

## Data Availability

The data presented in this study are available upon request from the corresponding author. The data are not publicly available due to privacy restrictions from FONDECYT 1200624 project
